# Serum Bile Acids in Repaired Tetralogy of Fallot: A Marker for Liver and Heart?

**DOI:** 10.1371/journal.pone.0144745

**Published:** 2015-12-11

**Authors:** Gernot Grangl, Evelyn Zöhrer, Martin Köstenberger, Alexandra Jud, Günter Fauler, Hubert Scharnagl, Tatjana Stojakovic, Robert Marterer, Andreas Gamillscheg, Jörg Jahnel

**Affiliations:** 1 Department of Pediatrics and Adolescent Medicine, Division of Pediatric Cardiology, Medical University Graz, Graz, Austria; 2 Clinical Institute of Medical and Chemical Laboratory Diagnostics, Medical University Graz, Graz, Austria; 3 Department of Radiology, Division of Pediatric Radiology, Medical University Graz, Graz, Austria; Innsbruck Medical University, AUSTRIA

## Abstract

**Background and Aims:**

Patients with repaired tetralogy of Fallot may develop chronic right ventricular dysfunction and hepatic congestion over time. We hypothesized that bile acid metabolism is altered in repaired tetralogy of Fallot patients and therefore sought to correlate right ventricular indices with serum bile acid levels.

**Methods:**

Indexed right ventricular end diastolic volume, as assessed by cardiac magnetic-resonance imaging, was classified as <100ml/m^2^ (Group 1, n = 5), 100–150ml/m^2^ (Group 2, n = 18), and >150ml/m^2^ (Group 3, n = 6) in 29 patients with repaired tetralogy of Fallot. Pulmonary regurgitation fraction and right ventricular ejection fraction were calculated. The serum bile acid profile, including 15 species, in these patients was determined by liquid chromatography coupled with mass spectrometry.

**Results:**

Serum bile acid levels increased from Group 1 to Group 3 (2.5 ± 0.7; 4.1 ± 2.5; 6.0 ± 2.8 μmol/l, respectively) with significantly increased bile acid values in Group 3 compared to Group 1 (p≤0.05). In Group 3, but not in Group 1 and 2, a significant increase in glycine-conjugated bile acids was observed. Pulmonary regurgitation fraction increased (12 ± 1; 28 ± 16; 43 ± 3%, Groups 1–3, respectively) and right ventricular ejection fraction decreased (48.4 ± 6.4; 48.5 ± 6.5; 42.1 ± 5.3%, Groups 1–3, respectively) with rising indexed right ventricular end diastolic volume.

**Conclusions:**

These preliminary results suggest that serum bile acid levels are positively correlated with indexed right ventricular end-diastolic volume in patients with repaired tetralogy of Fallot; however, this needs to be confirmed in a larger patient cohort.

## Introduction

Tetralogy of Fallot (TOF) is the most common form of cyanotic heart disease, accounting for 3.5% of congenital heart disease [1]. Surgical relief of right ventricular (RV) outflow tract obstruction can lead to pulmonary regurgitation (PR), which with age may cause progressive RV dilatation and dysfunction [2,3]. Assessing RV volume and function in these patients is of particular interest. Recently published indices for the assessment of RV function suggest that a significant volume overload leads to decreased systolic RV function in TOF patients, with increased indexed RV end-diastolic volume (RVEDVi), determined by magnetic resonance imaging [4–7]. Chronic RV dysfunction affects not only the cardiovascular system itself, but may lead to low cardiac output and tissue congestion, with liver dysfunction [8].

Liver function test result (LFTR) abnormalities are well characterized in adults with chronic heart failure [9,10]. Studies of cardiac hepatomegaly focus predominantly on LFTR changes in adults with chronic heart failure [11,12]. To the best of our knowledge, this is the first study to investigate bile acid (BA) metabolism in repaired TOF patients.

We hypothesized that liver congestion in patients with repaired TOF leads to alterations in BA metabolism and that serum BA levels may be useful markers of liver and right ventricular dysfunction in TOF patients. We determined serum BA levels using high-performance liquid chromatography—high-resolution mass spectrometry (HPLC-HRMS) and investigated the association of BA with imaging-study evidence of cardiac dysfunction.

## Patients and Methods

### Study Design and Patient Characteristics

We performed a prospective study comprising patients who after total surgical repair of TOF were routinely evaluated at the Division of Pediatric Cardiology, Medical University Graz, from January 2014 to March 2015. The study was approved by the Medical University Graz ethics committee (24–549 ex 11/12). Written informed consent to participate was obtained from the parents or legal guardians of the patients, or the patients themselves when aged >18 years. Blood samples were collected from patients after 12 hours of overnight fasting, with sera immediately separated and stored at -80°C until assays.

This study included 29 patients (18 female, 11 male) with surgically corrected TOF who were undergoing routine clinical follow-up. Some had participated in previous studies[6,7]. The RV outflow tract had been repaired by means of a transannular patch of autologous untreated pericardium in all patients. The patients were evaluated from the age of 11 to 43 years, at a time interval from 9 to 41 years after surgery. Anthropometric parameters were determined to calculate the body mass index (BMI). The patient group had a mild residual RV outflow tract gradient of 9 ± 5 mm Hg, as determined by echocardiography and MRI. The patients’ baseline characteristics are shown in Table 1. TOF patients with a higher degree of RV outflow tract obstruction, a valvular or pulmonary artery branch stenosis, and/or with restrictive physiology of the RV, defined as the presence of laminar antegrade diastolic main PA flow throughout the respiratory cycle by Doppler echocardiography, were excluded from the study. Additionally, patients with an implanted cardioverter-defibrillator, as unsuited to MRI, as well as patients with chromosomal disorders and primary liver or biliary-tract disorders were excluded. At the time point of blood sampling none of the patients took any relevant concomitant medication influencing right ventricular function and/or liver function. No relevant co-morbidities such as renal diseases or lipid metabolism disorders were found in our patients at routine follow-up examinations and routine blood testing. In all TOF patients RVEF and RVEDVi could be measured by MRI. The time interval between magnetic resonance imaging and laboratory analysis ranged from 0–14 days.

**Table 1:** Patient characteristics and laboratory parameters.

	Group 1 RVEDVi <100 ml/m^2^	Group 2 RVEDVi 100–150 ml/m^2^	Group 3 RVEDVi >150 ml/m^2^
**Number (total = 29)**	5	18	6
**Sex (f / m)**	3 / 2	11 / 7	3 / 3
**Age (years)**	32 ± 7	22 ± 7	24 ± 14
**BMI (kg/m** ^2^ **)**	21.6 ± 2.7	25.5 ± 5.4	20.3 ± 3.3
**RVEF (%)**	48.4 ± 6.4	48.5 ± 6.5	42.1 ± 5.4
**PRF (%)**	11.7 ± 1.2	27.7 ± 15.8	43.2 ± 3.2
**NT-pro BNP (pg/ml)**	161 ± 81	133 ± 125	262 ± 326
**ALT (U/l)**	23 ± 23	23 ± 10	32 ± 20
**AST (U/l)**	25 ± 6	24 ± 6	31 ± 10
**GGT (U/l)**	46 ± 46	27 ± 13	51 ± 25
**AP (U/l)**	57 ± 13	92 ± 60	95 ± 30
**tBA (μmol/l)**	2.5 ± 0.7 (0.4–4.7)	4.1 ± 2.5 (3.0–5.3)	6.0^a^ ± 2.8 (4.0–8.0)
**T-conjugates (μmol/l)**	0.1 ± 0.05 (0–0.7)	0.7 ± 0.7 (0.4–1.0)	0.4 ± 0.2 (0–1.0)
**G-conjugates (μmol/l)**	1.5 ± 0.7 (0–3.3)	2.8 ± 2.0 (1.8–3.7)	4.3^a^ ± 2.4 (2.7–6.0)

Values are expressed as mean ± SD. tBA are expressed as mean ± SD and 95% confidence interval. Expected ranges: BMI, 18.5–25 kg/m^2^; NT-pro BNP, <100 pg/ml; ALT, < 35 U/l; AST, < 43 U/l; GGT, < 38 U/l; AP, 35–105 U/l; tBA, 0.3–6.4 μmol/l; T-conjugates, 0.1–1.3 μmol/l; G-conjugates, 0.2–3.3 μmol/l.

^a^p≤0.05 compared with Group 1.

### Magnetic Resonance Imaging

RV volumes were quantified using breath-hold segmented gradient-recalled echo sequences obtained with a 1.5 T machine (Symphony; Siemens Medical Systems, Erlangen, Germany). Argus Software Numaris 4 (Siemens Medical Systems) was used for post-processing. The RV was encompassed by continuous short-axis views from base to apex. RV volumes were calculated after delineation of the endocardial borders in the end-diastolic and end-systolic images, with trabeculation assigned to the blood pool [13]. Also a delayed contraction of the right ventricle due to a right bundle branch block was considered in the evaluation of RV function [14]. RVEDVi > 150 mL/m^2^, defined from MRI data reported for adults and adapted for children, corresponded to 150% of the normal upper limit for RV end-diastolic volume in children (100 mL/ m^2^) [15]. Pulmonary regurgitation (PR) was quantified by velocity-encoded imaging. The PR fraction (PRF) was determined by calculating the percentage of reverse volume from gross forward volume. All volumes and flow measurements were indexed for body surface area and expressed in milliliters per beat per meters squared. A pediatric radiologist blinded to echocardiographic data did all the measurements.

### Laboratory Analysis

Analytes included, as LFTR, serum activities of alanine transaminase (ALT), aspartate transaminase (AST), gamma-glutamyl transpeptidase (GGT), alkaline phosphatase (AP), and, as a marker for cardiac insufficiency, concentrations of N-terminal pro-brain natriuretic peptide (NT-proBNP). In addition, total BA (tBA) levels were measured by HPLC-HRMS, including unconjugated and taurine (T)- and glycine (G)- conjugated species. A full 15-BA profile was determined using 10 μl of serum; it included cholic acid (CA), chenodeoxycholic acid (CDCA), deoxycholic (DCA), lithocholic (LCA), and ursodeoxycholic (UDCA) acid and their T-conjugates TCDC, TLCA, TDCA, TCA, and TUDCA and G-conjugates GCA, GCDC, GLCA, GDCA, and GUDCA. Individual BA were separated by HPLC using a reversed-phase C18 column with a methanol and water gradient and deuterium-labeled internal standards. Mass spectrometer Q Exactive^™^ MS/MS (Thermo Fisher Scientific, Waltham, MA) and high-performance quadrupole precursor selection with high-resolution and accurate-mass (HR/AM) Orbitrap^™^ detection permitted sensitive quantitation (Amplatz *et al*., submitted).

### Statistical Analysis

Patient characteristics and laboratory parameters are reported as mean and standard deviation (SD). Groups were compared using univariate ANOVA. Correlations between BA and cardiac parameters were assessed using Pearson's correlation analysis. All statistical tests were two-tailed and p values of <0.05 were considered as statistically significant. The SPSS Statistics package 21.0.0 (IBM SPSS, Armonk, NY) was used for all analyses.

## Results

### Serum BA levels, LFTR, and cardiac insufficiency marker values in patients with RV dilatation

**Fig 1:**
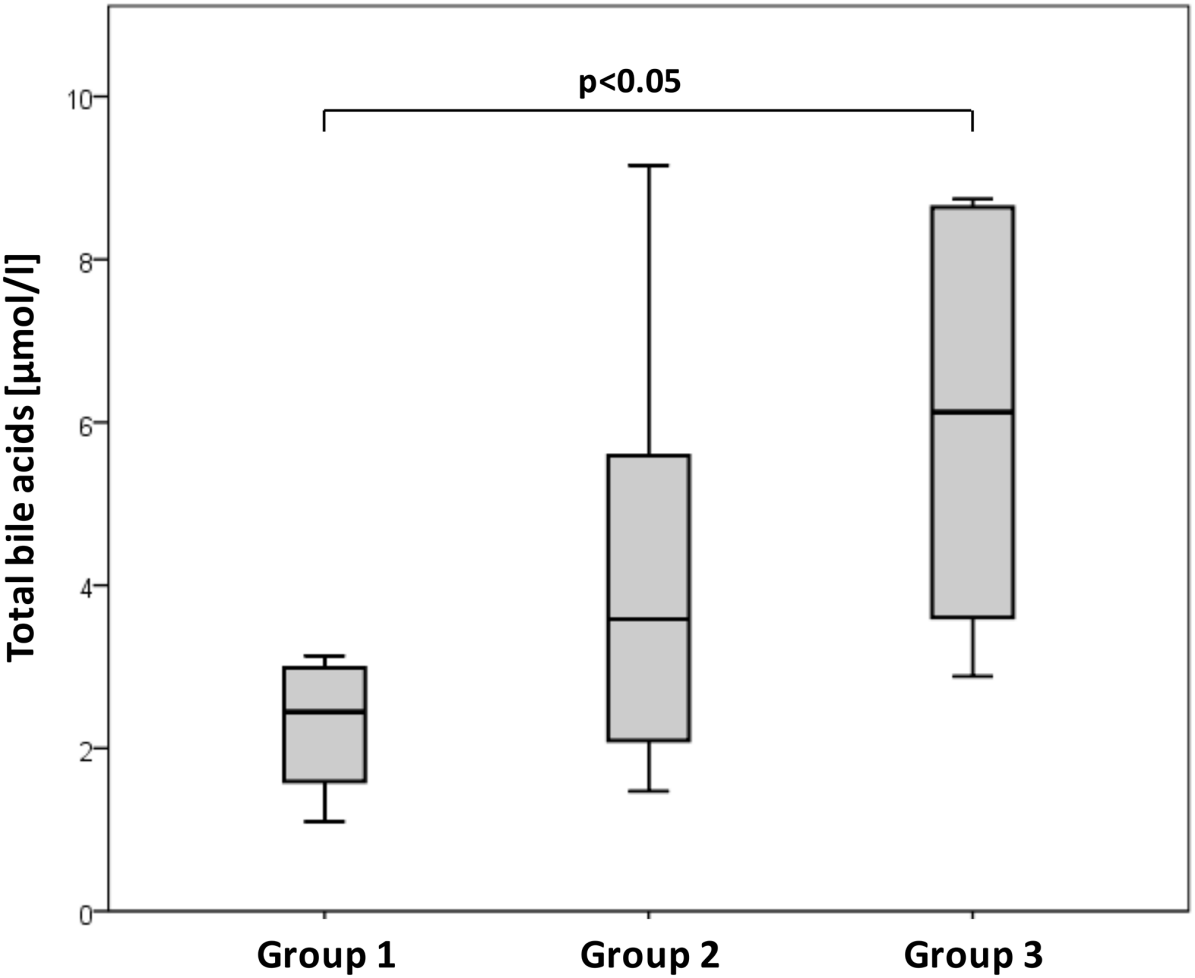
Total BA levels in TOF patients with RVEDVi (1) <100 ml/m^2^, (2) 100–150 ml/m^2^, and (3) >150 ml/m^2^. BA levels were significantly elevated in Group 3 compared to Group 1 (p≤0.05). Total BA levels rose in TOF patients with increasing RVEDVi.

Twenty-nine TOF patients were stratified into 3 groups according to grade of RV dilatation: 1) RVEDVi <100 ml/m² (no significant dilatation; n = 5; males: females = 2: 3; age 32 ± 7 years), 2) RVEDVi 100–150 ml/m² (mild-moderate dilatation; n = 18; males: females = 7: 11; age 22 ± 7 years) and 3) RVEDVi >150 ml/m² (severe dilatation; n = 6; males: females = 3: 3; age 24 ± 14 years). Mean levels of serum BA at 2.5 ± 0.7 μmol/l (0.4–4.7) were slightly, but not significantly lower in Group 1 than in Group 2 (4.1 ± 2.5 μmol/l, 3.0–5.3). However, in Group 3 mean BA levels were significantly elevated (6.0 ± 2.8 μmol/l, 4.0–8.0; p≤0.05) compared to Group 1 (Fig 1). Serum activities of ALT, AST, GGT, and AP were within expected ranges in all 3 groups, although ALT and AST values were higher in Group 3 patients than in Group 1 (23 ± 23 U/l and 25 ± 6; 23 ± 10 U/l and 24 ± 6; 32 ± 20 U/l and 31 ± 10 in Groups 1, 2, and 3, respectively). Mean NT-proBNP values were above the upper limit of normal in all 3 groups (normal range < 100pg/ml) and rose together with RV dilatation (161 ± 81 pg/ml, 133 ± 125 pg/ml, and 262 ± 325 pg/ml in Groups 1, 2, and 3, respectively). Values for all parameters are listed in Table 1.

### BA profile in patients with repaired TOF

**Fig 2:**
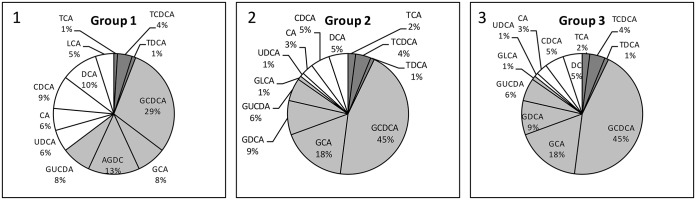
BA pool composition in patients with RVEDVi (1) <100 ml/m^2^, (2) 100–150 ml/m^2^, and (3) >150 ml/m^2^. In Group 2 T-conjugated and G-conjugated BA were increased compared to Group 1. In Group 3 T-conjugates were increased compared to Group 1, but decreased compared to Group 2. G-conjugates were significantly increased in Group 3 compared to Group 1 (p≤0.05). GCDCA was the predominant BA in all 3 groups, rising with increasing RVEDVi. White: unconjugated BA, light grey: G-conjugated BA, dark grey: T-conjugated BA. Abbreviations: BA, bile acids; G, glycine; T, taurine; RVEDVi, right ventricular end-diastolic volume.

A full BA profile was determined in all 3 groups (Fig 2). Tauro-conjugates were increased in Group 2 (0.7 ± 0.7 μmol/l, 0.4–1.0) and Group 3 (0.4 ± 0.2 μmol/l, 0–1.0) compared to Group 1 (0.1 ± 0.1 μmol/l, 0–0.7). In all 3 groups TCDCA and TCA were the 2 most abundant T-conjugated BA. Both were significantly increased in Group 3 compared to Group 1 (p≤0.01). Glyco-conjugates increased together with increasing RV dilatation (1.5 ± 0.7 μmol/l, 0–3.3; 2.7 ± 2.0 μmol/l, 1.8–3.7; and 4.3 ± 2.4 μmol/l, 2.7–6.0, in Groups 1, 2, and 3 respectively); levels were significantly increased in Group 3 compared to Group 1 (p≤0.05). GCDCA and GCA, the two main G-conjugated BA, were significantly increased in Group 3 compared to Group 1 (p≤0.05). In addition, primary BA fractions rose as RV dilatation increased (57%, 69%, and 75% in Groups 1, 2, and 3 respectively).

### Correlation of BA values with RVEDVi values

**Fig 3:**
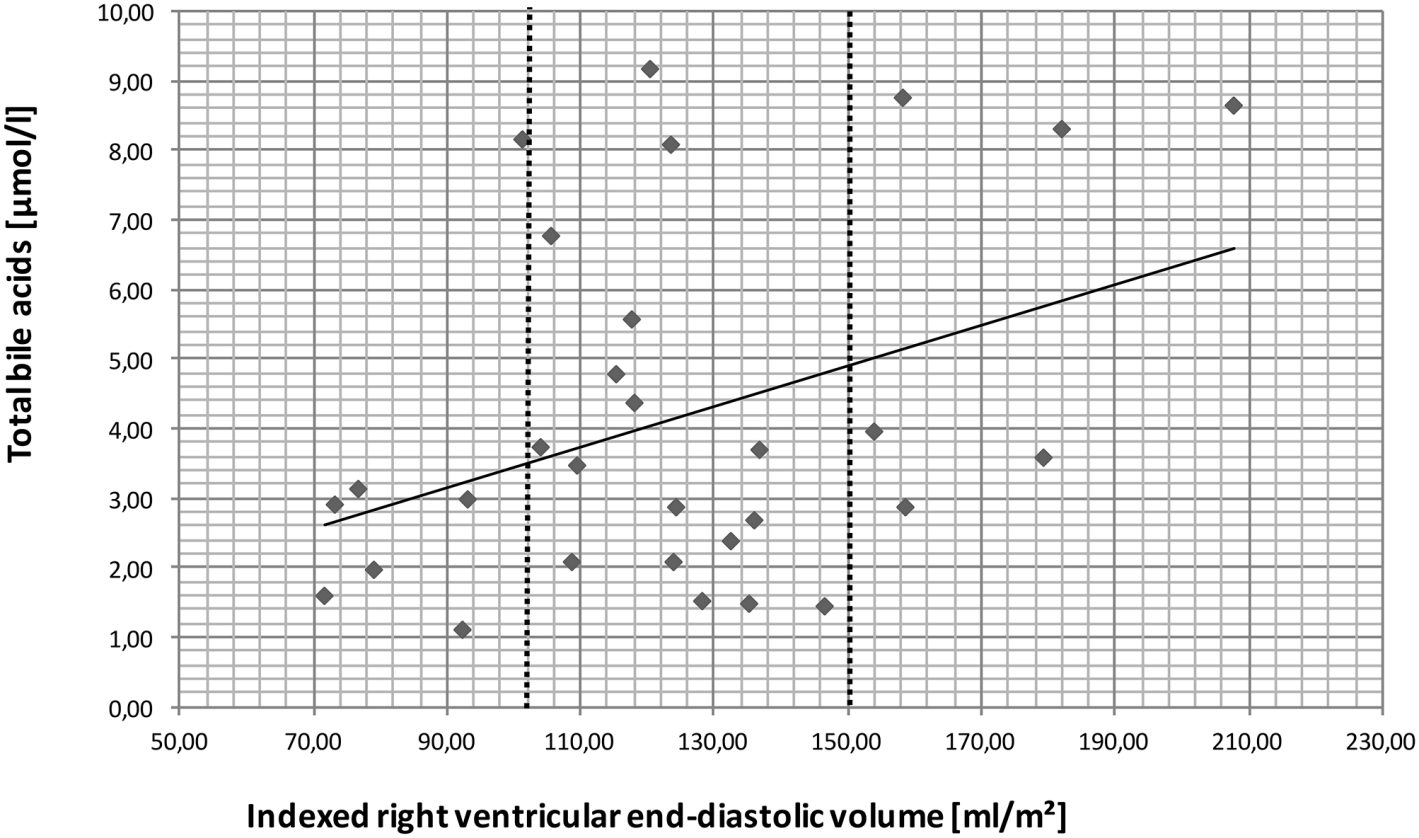
Correlation of total BA levels with RVEDVi. Together with increasing RVEDVi, total BA values also increased (r = 0.4; p≤0.05). *Abbreviations*: BA, bile acids; RVEDVi, indexed right ventricular end-diastolic volume.

RVEF decreased with increasing RVEDVi from Group 1 to Group 3 (48.4 ± 6.4%, 42.1 ± 5.3% in Groups 1, 3, respectively). PRF increases were considered to reflect late complications in ToF; in Group 1 PRF was low at 11.7 ± 1.2%, in Group 2 it was moderate at 27.7 ± 15.8%, and in Group 3 it was moderate to high at 43.2 ± 3.2%. Values for all parameters are listed in Table 1. A significant negative correlation existed between RVEF and RVEDVi (r = -0.4; p≤0.05) as a marker of RV dysfunction and RV dilatation; and a significant positive correlation existed between BA levels and RVEDVi (r = 0.4; p≤0.05) (Fig 3).

## Discussion

After TOF repair, progressive RV dilatation and dysfunction related to PR are an important clinical concern [16,17]. Such RV dilatation and dysfunction may induce liver congestion and therefore damage. Our study aimed to determine BA levels in our repaired TOF patients to investigate if BA might serve as a sensitive marker for liver damage that was not reflected by conventional LFTR.

Tetralogy of Fallot (TOF) is the most common form of cyanotic congenital heart disease [18]. Although the survival of patients with TOF has improved considerably, late complications, including PR, are common. PR causes volume overload of the RV, which can lead to RV dilatation and dysfunction that end in right heart failure [19]. An analyte commonly used to detect early changes in RV function is NT-proBNP, a marker of increased myocardial-wall stress. Our study found that TOF patients with increasing RV dilatation showed an increase in NT-proBNP levels. This is in accordance with a study from Eindhoven *et al*. in 2014 [20], who demonstrated the potential of NT-proBNP to monitor ventricular function in TOF patients, with timely detection of clinical deterioration. Chronic RV volume overload and eventually RV ventricular failure may lead to congestive hepatomegaly as proposed by Ford *et al*. in 2015[21]. Congestive hepatomegaly is often associated with impaired hepatic function. Its progress is routinely evaluated using classical LFTR, including serum ALT, AST, GGT, and AP activities.

Changes in BA values can track hepatic and intestinal disorders. BA are a group of steroids representing the end products of cholesterol metabolism. Two primary BA, CA and CDCA, are synthesized principally in hepatocytes, with conjugation to amino acids G and T. DCA, LCA, and UDCA, which originate from CA and CDCA that have undergone deconjugation and dehydroxylation by various gut bacteria, are considered secondary BA [22]. BA reabsorbed through the intestinal wall into the portal circulation are taken up by the liver, and BA levels in hepatic venous effluent (and thus in peripheral blood) are normally low [23].

Total BA levels were within expected ranges in our TOF patients (reference values from healthy adults, 0.3–6.4 μmol/l) [24]. However, total BA levels rose in our TOF patients with increasing RV dilatation as indicated by RVEDVi. BA values were significantly elevated in Group 3 patients (with high RVEDVi values) compared to Group 1 patients (with low RVEDVi values). Group 1 and Group 3 BMIs were within expected ranges; patients in Group 3 had a slightly elevated mean BMI. Elevated transaminase values are sensitive markers of liver cell injury. Interestingly, in our study ALT and AST values were slightly elevated in Group 3 compared to Group 1; however, they were within normal ranges. In addition, GGT and AP were within normal ranges in all patients, suggesting biliary tract integrity.

Although conventional LFTR indicated no hepatobiliary injury, we found a significant difference in primary BA values between patients in Group 1, with minor RV dilatation, and patients in Group 3, with significant RV dilatation. Serum BA might increase due to increasing spillover into the blood caused by a reduced efflux via the RV or due to reduced biliary BA flow into the gut. Therefore the enterohepatic circulation (EHC) of BA might be modified. EHC is regulated by the nuclear receptor for BA, the farnesoid X receptor (FXR), and by fibroblast growth factor-19 (FGF-19) levels in liver and gut [25,26]. Deficient FXR activation in the gut due to decreased intraluminal BA levels frees BA *de novo* synthesis in the liver. In summary, BA synthesis might be stimulated via FXR-mediated feedback prompted by diminished intestinal BA levels, eventually leading to higher proportions of primary BA.

Not only total serum BA levels vary in repaired TOF patients, but also the proportions of individual BA, as depicted in Fig 1. A high RVEDVi was associated with a higher percentage of conjugated BA, with a significant increase in G-forms (1.5 ± 0.7 μmol/l, 2.7 ± 2.0 μmol/l, and 4.3 ± 2.4 μmol/l in Groups 1, 2, and 3 respectively; p≤0.05). G-conjugates of Group 3 were above expected ranges (0.2–3.3 μmol/l) [24]. Interestingly, T-conjugate levels increased from Group 1 to Group 2, tracking rises in RVEDVi, but decreased in Group 3, where RVEDVi was highest (0.1 ± 0.05 μmol/l, 0.7 ± 0.7 μmol/l, and 0.4 ± 0.2 μmol/l in Groups 1, 2, and 3 respectively). Compared to healthy adult controls, T-conjugates were within normal ranges in all 3 groups (0.1–1.3 μmol/l) [24].

## Conclusion

To the best of our knowledge, this is the first report that peripheral-blood BA levels are positively correlated with increasing RV dilatation. Our results indicate that determination of serum BA levels may be of diagnostic value in patients with repaired TOF, with sensitivity to hepatobiliary injury greater than that of usual LFTR. This information is of value in understanding the pathogenesis and potential treatment of repaired TOF patients at risk of congestive liver disease; however, the clinical significance of shifts in circulating BA concentrations is not established, and cardiac MRI will remain the gold standard in determining cardiac function in repaired TOF. The major limitation of this study is the relatively small numbers of patients studied, due to the single-center study design. At this moment we cannot exclude the possibility that the patient population enrolled in this study is biased toward less significant residual lesions after TOF repair. Nevertheless, determination of BA may offer a sensitive approach to identify early hepatobiliary injury in repaired TOF patients due to a deterioration of RV function, particularly when other LFTR are not recognizably abnormal. This possibility awaits clarification in larger cohorts.

## Abbreviations

TOF: Tetralogy of Fallot
PR: pulmonary regurgitation
RV: right ventricular
RVEDVi: indexed RV end-diastolic volume
LFTR: liver function test result
MRI: magnetic resonance imaging
BA: bile acids
HPLC-HRMS: high-performance liquid chromatography—high-resolution mass spectrometry
BMI: body mass index
PRF: pulmonary regurgitation fraction
ALT: alanine transaminase
AST: aspartate transaminase
GGT: gamma-glutamyl transpeptidase
AP: alkaline phosphatase
NT-proBNP: N-terminal pro-brain natriuretic peptide
tBA: total BA
T: taurine
G: glycine
CA: cholic acid
CDCA: chenodeoxycholic acid
DCA: deoxycholic acid
LCA: lithocholic acid
UDCA: ursodeoxycholic acid
SD: standard deviation
